# Tele-education in point-of-care ultrasound training

**DOI:** 10.1186/s13089-024-00394-1

**Published:** 2024-10-28

**Authors:** Reina Suzuki, William J. Riley, Matthew S. Bushman, Yue Dong, Hiroshi Sekiguchi

**Affiliations:** 1 Division of Pulmonary and Critical Care Medicine, Mayo Clinic, Rochester, MN USA; 2 Healthcare Technology Management, Mayo Clinic, Rochester, MN USA; 3 Center for Digital Health, Mayo Clinic, Rochester, MN USA; 4 Department of Critical Care Medicine, Mayo Clinic, 5777 East Mayo Blvd, Phoenix, AZ 85054 USA; 5 Department of General Internal Medicine, Jichi Medical University Saitama Medical Center, Saitama, Japan

**Keywords:** Ultrasound, Point-of-care ultrasonography, Critical care, Tele-education, Remote coaching

## Abstract

**Background:**

Traditionally, ultrasound skills have been taught through a one-on-one approach, where instructors physically guide learners’ hands at the bedside or in the workshop. However, this method is frequently challenged by scheduling and cost limitations. Our objective was to create a tele-education model for point-of-care ultrasound training and evaluate its effectiveness and its impact on learners’ perceived workload compared to conventional education and self-directed learning methods.

**Methods:**

We conducted a 3-arm randomized trial, comparing tele-education (TE), conventional education (CE) and self-directed learning (SL) methods. All subjects underwent online didactic lectures prior to a hands-on ultrasound workshop. The TE group utilized an ultrasound machine equipped with a speakerphone, a webcam for direct visualization of learner’s hand maneuvers, and an analog-to-video converter for the real-time streaming of ultrasound images. This configuration enabled remote instructors to provide immediate verbal feedback to learners. In contrast, the CE group received in-person coaching, while the SL group had no instructors present. Following the coaching session, subjects completed a scenario-based skill test and a survey on the National Aeronautics and Space Administration task load index (NASA-TLX) to measure their ultrasound competency and perceived workload, respectively.

**Results:**

Twenty-seven ultrasound novices were randomly allocated into 3 groups. The median skill test score of TE, CE, and SL was 22 [interquartile range (IQR): 18–28], 24 [IQR: 21–31], and 16 [IQR: 15–18], respectively (*p* < 0.01). Pairwise comparisons of median test scores of 3 groups demonstrated a statistical significance in comparisons of TE vs. SL (22 vs. 16, *p* = 0.01) and CE vs. SL (24 vs. 16, *p* < 0.01), but not in TE vs. CE (22 vs. 24, *p* = 0.56). There was no statistical significance observed in the median NASA-TLX scores among the 3 groups; 54 [IQR:47–61] in TE, 57 [IQR:22–64] in CE, and 66 [IQR: 66–72] in SL (*p* = 0.05).

**Conclusions:**

Our tele-education model was more effective than self-directed learning. There was no statistically significant difference in effectiveness between the tele-education and the conventional education groups. Importantly, tele-education did not impose a significantly higher workload on learners compared to conventional education or self-directed learning. Tele-education has a substantial potential as an alternative to conventional ultrasound training.

**Supplementary Information:**

The online version contains supplementary material available at 10.1186/s13089-024-00394-1.

## Introduction

Point-of-care ultrasonography (POCUS) is an important adjunct to physical examination and bedside procedures [1–3]. There have been an increasing number of reports that POCUS can modify diagnoses, direct further testing, and change medical therapy [4–12]. On the other hand, it is well known that ultrasonography is a highly operator-dependent examination [13, 14]. Multiple steps are required for its safe and effective usage in patient care, such as image acquisition, image interpretation, and information integration into clinical decisions. Specifically, image acquisition is the most important skill to have, since subsequent steps are based on the assumptions that the optimal images have been obtained. Traditionally, image acquisition skill has successfully been taught in a one-on-one approach, where on-site instructors directly guide learners in machine operation, transducer orientation, and hand maneuvers in a workshop or a seminar [15, 16]. This method, however, has confronted various barriers such as the difficult scheduling of learners and instructors, the relative shortage of instructors, and the cost of equipment and workshop organization [17–20]. A multimedia workshop combining online didactic modules and hands-on training sessions has the potential to ease some of those barriers; [15, 16] yet, the travel cost to attend a regional workshop still remains to be a challenge for both learners and instructors [17]. 

These potential challenges are not unique to POCUS education; in fact, they seem to be rather universal and common in medical educational fields. With advances in technology and interconnected media, and necessitated by the recent COVID-19 pandemic which forced many in-person activities to be converted to online-based programs, an increasing number of medical organizations have started to use tele-education in an attempt to eliminate those barriers [21–23]. There have been several pilot studies that described the concept of remote education or remote tele-mentored ultrasound in POCUS [24–29]. Specifically, during the COVID-19 pandemic, a few studies described novel tele-ultrasound curricula in medical schools that incorporated tele-mentoring on image acquisition through a recently released sophisticated tele-ultrasound software [27, 28]. In these curricula, ultrasound knowledge was learned via Web-based didactic lectures. Although these studies suggested several advantages (resolutions of physical constraints, no cost for traveling), and disadvantages (technical limitations, lack of hand maneuver guidance, cognitive difficulty of learners in remote guidance), there have only been a few clinical studies that evaluated the effectiveness of remote POCUS coaching on learners’ image acquisition skill compared to those of conventional instructor-led hands-on training or self-directed learning [27, 28]. Furthermore, there has been little description of quantitative learners’ workload or cognitive stress in remote coaching compared to conventional training or self-directed learning.

In the present study, we first developed a practical, but HIPAA-compliant secure tele-education system that could be applied not only in ultrasound education but also in the actual patient care. We also developed online pre-training didactic lectures including the video demonstration of ultrasonography examinations that would accelerate leaners’ understanding of POCUS. Subsequently, we aimed to evaluate the effectiveness of this tele-education model in a prospective 3-arm randomized trial, by comparing it to (1) the conventional hands-on education and (2) the self-directed learning without an instructor. Furthermore, we sought to describe learners’ perceived workload associated with those 3 training models. Our hypothesis was that tele-education is more effective than self-directed learning and comparable to the conventional hands-on education in improving learners’ image acquisition skills. We also hypothesized that learners’ perceived workload in the tele-education training is greater than that of conventional education but less than that of self-directed learning group.

## Methods

### Setting

We conducted a prospective, 3-arm randomized trial to compare 3 different POCUS education methods (tele-education, conventional, and self-directed learning) in learners’ improvement in POCUS skill proficiency and training workload in an academic teaching institution (Mayo Clinic, MN, USA) from May through June, 2016. The Mayo Clinic Institutional Review Board approved the study [study ID: 15-007311].

### Study participants

Ultrasound novices were recruited among the groups of physicians, nurse practitioners, physician assistants, and medical students in the Mayo Graduate School of Medicine and the Mayo Clinic Health System. Novices were defined as individuals who have not conducted POCUS in the actual patient care except for ultrasound-guided central line insertions. Individuals who previously attended a regional workshop were excluded from the study. Verbal consent for study participation and videotaping was obtained from all study participants.

### Study design

**Fig. 1:**
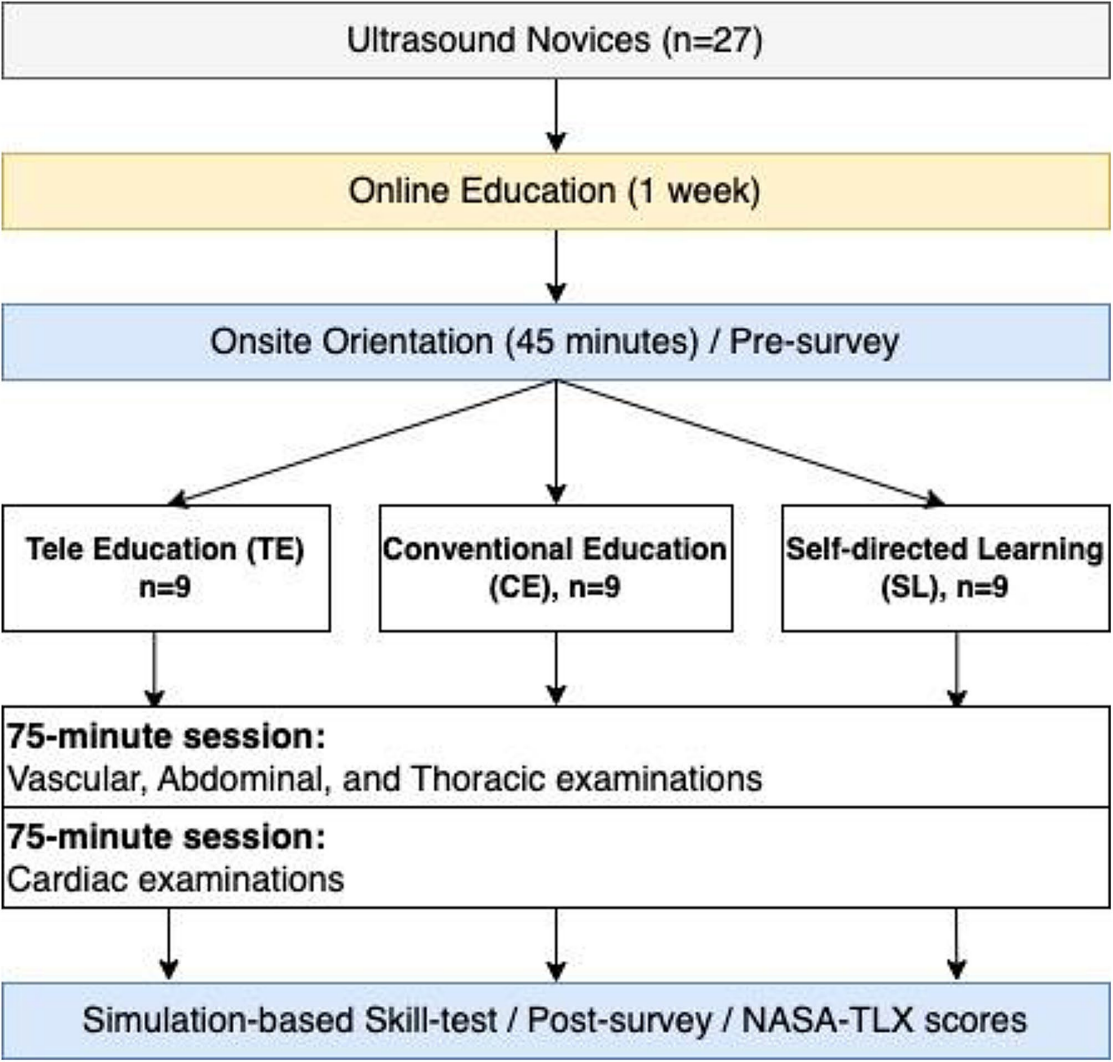
Study design

The study design is summarized in Fig. 1. After verbal consent was obtained from study participants, they were randomized into one of the 3 education arms. A group allocation was concealed from participants until the start of the hands-on training session. One week prior to the scheduled hands-on training date, participants were given access to online didactic lectures on POCUS basics, consisting of mandatory (80 min) and optional lectures (140 min). These online materials were posted on the dedicated YouTube channel, and the participants were encouraged to review them as many times as they wished. Online lectures consisted of 14 video modules that aimed to establish the basics of ultrasound physics, knobology, image acquisition, and image interpretation. On a hands-on training date, participants were asked to attend a 30-minute orientation session in a tele-conference format. During the orientation session with the use of PowerPoint via Zoom teleconference (CA, USA), a remote instructor reviewed the ultrasound machine and training room settings, standardized cues for ultrasound transducer orientation and hand maneuver, specific goals of the training, and a brief explanation of National Aeronautics and Space Administration task load index (NASA-TLX) survey (Supplementary Material 1). All questions from participants were answered during this session. At the end of the session, the group allocation was announced to the participants. All participants underwent two 75-minute POCUS hands-on training on a healthy male standardized patient in an assigned training method. Knobology, vascular, lung, and abdominal POCUS examinations were covered in the first 75 min of the hands-on session, and the latter 75 min were dedicated to cardiac POCUS. This training session was followed by a 30-minute post-training skill test (Supplementary Material 2) [30] and the NSAS-TLX survey to assess participants’ perceived workload. Optional conventional instructor-led hands-on sessions were offered for tele-education and self-directed learning groups after the completion of the skill tests for their own education.

### Three hands-on training methods; tele-education, conventional education, and self-directed learning

A hands-on examination room in each training method was equipped with anatomy models of the heart, the kidney, and the right upper quadrant abdominal organs, a computer with access to online didactic lectures, and an ultrasound machine (M-turbo, FUJIFILM SonoSite, Washington, USA) with 3 different transducers (linear, curvilinear, and phased array). A trainee: instructor ratio was kept 3:1 except a self-directed learning group, where no instructor was available.

#### Tele-education method

**Fig. 2:**
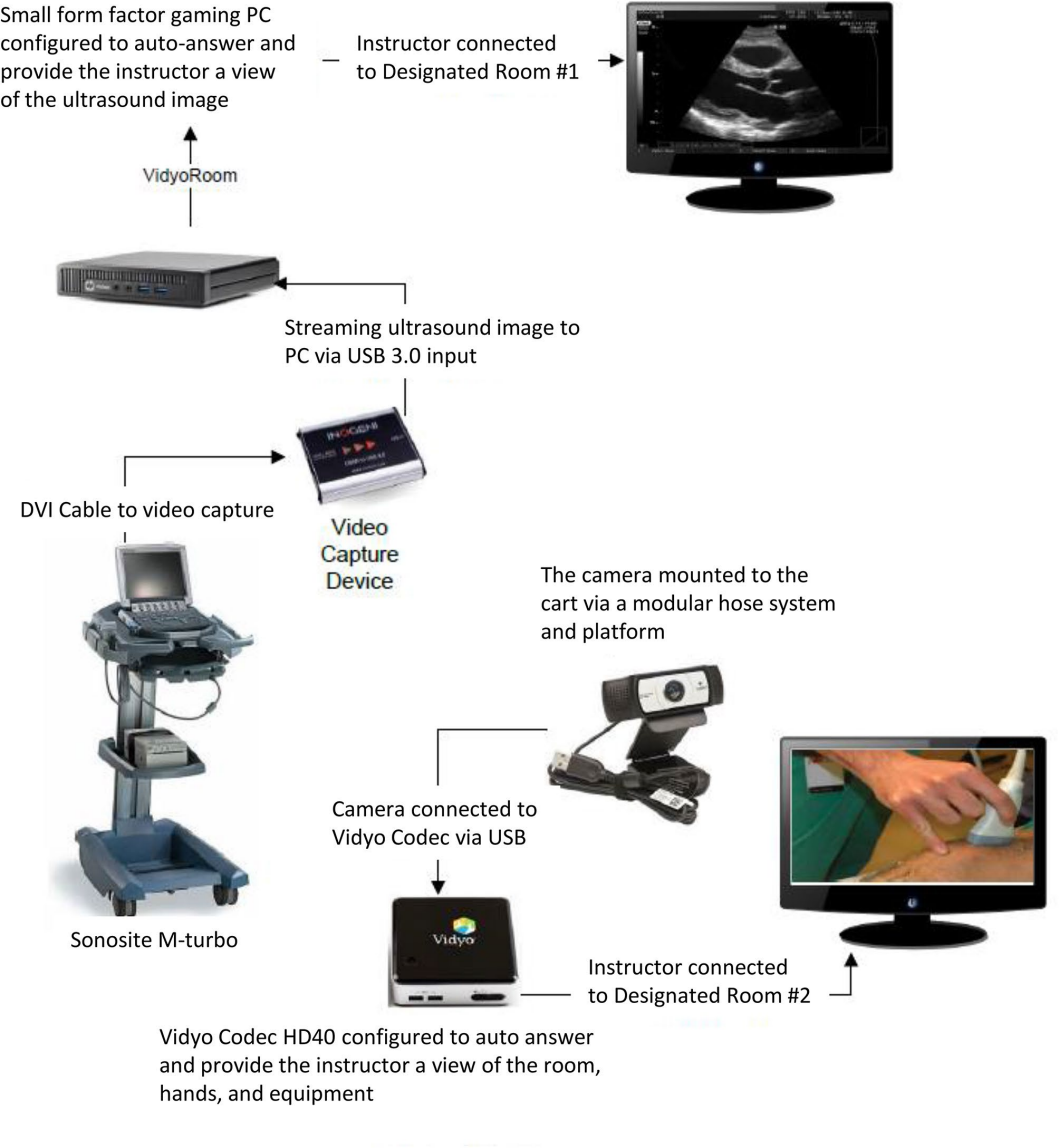
System configuration of the 2-way communication system

**Fig. 3:**
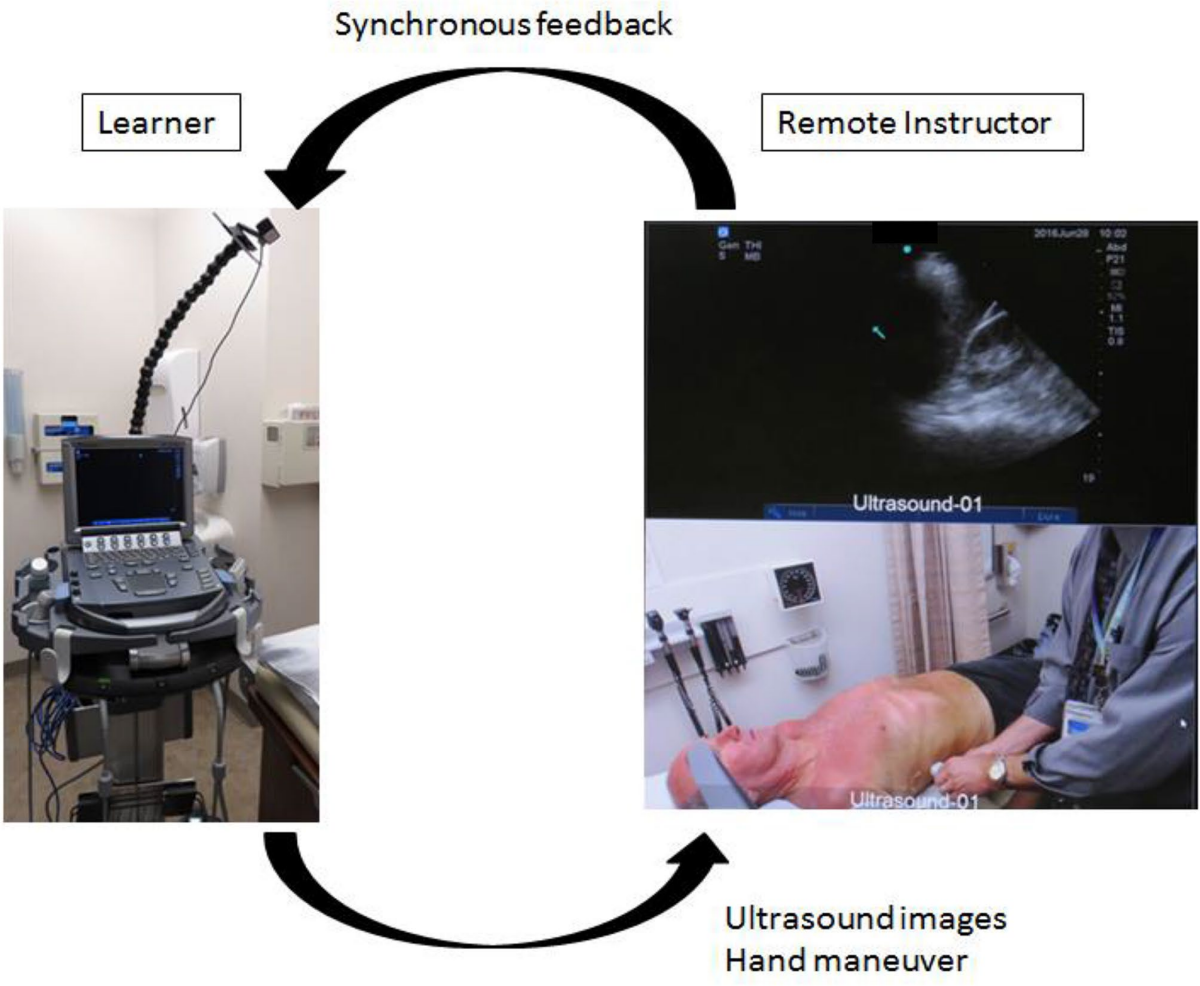
Appearance of the 2-way communication system. (Fig. 3. Legends) Left: An ultrasound cart placed in the learners’ room. The cart featured essential components, including a speakerphone, a webcam mounted on a flexible modular hose for direct visualization of learner’s hand maneuvers, and an analog-to-video converter, which enabled the real-time streaming of ultrasound images. This configuration allowed learners to receive immediate verbal feedback from the remote instructor via the speaker. Right: The instructor’s view. The real-time verbal feedback was provided to the learner based on dual video feeds: one from the ultrasound machine and the other from the webcam capturing the learner’s hand maneuvers

An instructor verbally guided learners’ machine operation and hand maneuver using a 2-way remote communication system attached to the ultrasound cart (Figs. 2 and 3). The remote coaching system consisted of 4 key components (1) a speakerphone (DUET PCS – MT202-PCS, California, USA) mounted directly to the ultrasound cart, (2) a Logitech c930e Webcam (California, USA) mounted to an adjustable modular hose to allow direct visualization of learners’ hand maneuvers, (3) INOGENI analog-to-digital video converter (Quebec, CAN) to enable real-time streaming of the ultrasound images to Vidyo video conference solution via (4) Vidyo Room HD40 codec (New Jersey, USA) to allow for Health Insurance Portability and Accountable Act (HIPAA) compliant system. The entire system was configured to auto-activate once the ultrasound machine was turned on. In addition to audio communication, dual video feeds on the Vidyo platform were available on the remote instructor’s computer. All the video/audio interactions occurred on the Vidyo platform and behind the secure firewall of Mayo Clinic Intranet through a hard-wired connection.

#### Conventional education method

An on-site instructor taught 3 novice leaners in the examination room. The instructor guided learners’ machine operation and hand maneuver. Anatomical models and video lectures were used as necessary to increase learners’ understanding.

#### Self-directed learning method

No instructor was available, and participants were encouraged to view pre-course materials online with a computer available in the room and help one another to facilitate their learning experience. The participants were also allowed to use the anatomic models available in the room.

### Outcome measurements

Our primary outcome was POCUS skill test scores. We utilized the scenario-based skill assessment of our POCUS proficiency test that was used in our previous works [15, 16, 30]. The assessment consisted of 6 sections to evaluate the exam initiation, vascular POCUS, lung POCUS, abdominal POCUS, cardiac POCUS, and the exam completion. Each section was timed by a study facilitator. The participants were asked to save video clips of scenario-based tasks documented in the instruction sheets. The saved video clips were subsequently reviewed and scored by one of the investigators on a checklist developed by the study team. The checklist was utilized in our previous work which demonstrated good concordance between two score raters [30]. 

Our secondary outcomes were improvement in learners’ confidence levels and perceived workload associated with each training method. Pre-training survey collected the participants’ confidence levels in machine operation, image acquisition, and image interpretation (all out of 10), and time spent on pre-course learning. Post-training survey consisted of the same questions on confidence levels as in the pre-training survey and 4 additional questions on course improvement and satisfaction on each training method they were allocated. Tele-education group was asked to answer 2 further questions: one inquiring about personal preferences on remote tele-education vs. conventional on-site guidance on a scale of 1–10 “Do you think that the quality of your ultrasound training would have been better if the instructor was present in the room?”, and the other inquiring agreement to the sentence “I would recommend the remote ultrasound training to my colleagues” on the scale of 1–10. The post-training and the NASA-TLX surveys were completed immediately after the training session before the skill test conduction.

### Sample size calculation and statistical analysis

All continuous data were presented with the median and interquartile ranges (IQRs). The time spent on pre-learning modules were presented with the number of participants in each category (% in each group), and the Fisher’s exact test was conducted with a web applet available at the following link at the time of protocol preparation: http://www.physics.csbsju.edu/stats/exact_NROW_NCOLUMN_form.html.

All continuous variables were tested with Kruskall-Wallis tests followed by pairwise comparisons among the 3 groups with 2-sample t-tests with a corrected alpha level of 0.017 (0.05 divided by 3). Otherwise, a p-value less than 0.05 (two-sided) was considered as statistically significant.

The target sample size was calculated as 18 in total, based on the results in our previous studies where POCUS proficiency of novice leaners was measured with the same assessment tools we utilized in this study. Assuming a standard deviation of 10% and an anticipated difference of 20% in terms of skill-test scores for a two-sample t-test, six participants per each group allocation was considered enough to achieve more than 80% power with an alpha level of 0.05. All the statistical analyses except for Fisher’s exact test were conducted with JMP 10 (SAS, Cary, USA).

## Results

Twenty-seven participants (18 nurse practitioners, 8 physicians, and 1 medical student) were randomly allocated into the tele-education (*n* = 9), conventional education (*n* = 9), or the self-directed learning group (*n* = 9). Baseline characteristics of ultrasound-novice participants, including subjective confidence levels in machine operation, image acquisition, image interpretation on a 10-point Likert scale, and the time spent on online pre-course materials are summarized in Table 1. There was no statistically significant association observed between subjective confidence levels and time spent on online pre-course materials among the groups.

**Table 1:** Baseline characteristics of tele-education (TE) vs. conventional education (CE) vs. self-directed learning (SL) groups

	TE [IQR]	CE [IQR]	SL [IQR]	*P*-value
Time spent on pre-course materials				
0–1 h	1	0	1	0.24
1–2 h	7	4	5	
2–3 h	1	5	2	
> 3 h	0	0	1	
Confidence levels				
Machine operation	2 [1–3]	3 [2-3.5]	3 [1–3]	0.41
Image acquisition	2 [1–3]	3 [1–3]	1 [1-2.5]	0.58
Image interpretation	1 [1-2.5]	2 [1–3]	2 [1-3.5]	0.55

The median [IQR] values of the following parameters are presented in Table 2; post-training skill test score (maximum of 50), improvement in participants’ subjective confidence levels, and NASA-TLX score.

**Table 2:** Results

	TE [IQR]	CE [IQR]	SK [IQR]	*P*-value
Skill test scores	22 [18–28]	24 [21–31]	16 [15–18]	< 0.01
Improvement in Confidence levels				
Machine operation	5 [4-5.5]	5 [3.5-5]	2 [1.5-3]	< 0.01
Image acquisition	5 [3-5.5]	4 [3-5.5]	2 [2-3.5]	< 0.01
Image interpretation	5 [4–6]	4 [2.5–5.5]	2 [1–3]	< 0.01
NASA-TLX scores	54 [47–61)	57 [22–64]	66 [63–72)	0.05

**Fig. 4:**
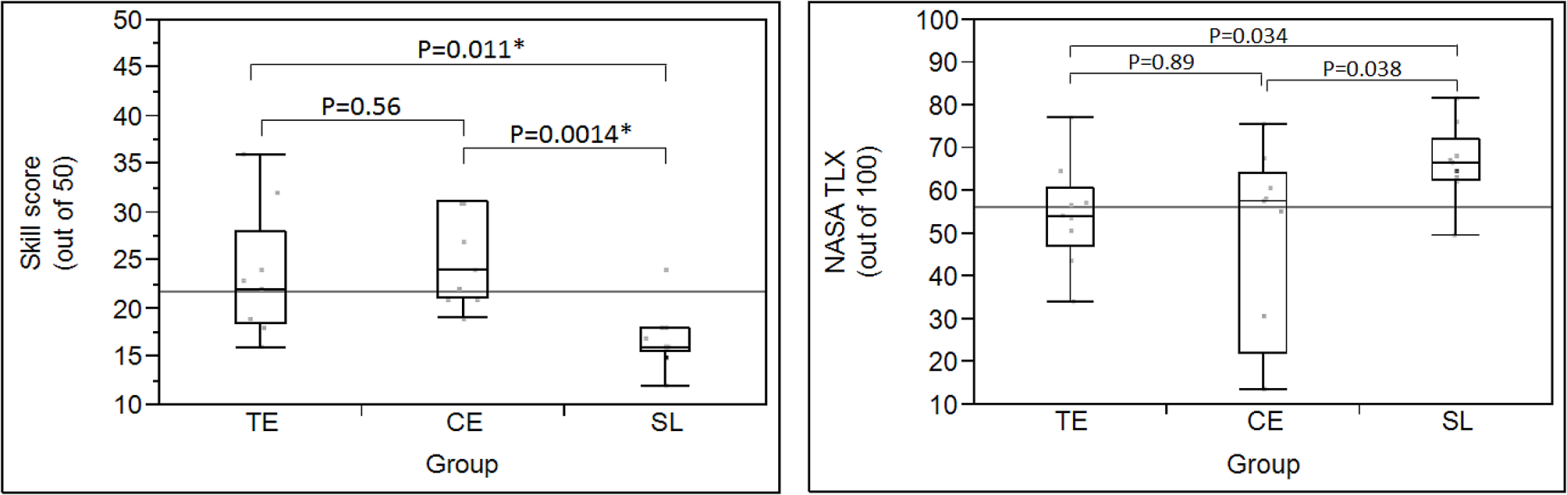
Proficiency test scores and NASA-TLX scores. Left: Proficiency test scores of the 3 groups. Right: NASA-TLX scores of the 3 groups. TE: tele-education, CE: conventional education, SL: self-directed learning

There was a significant difference between the median post-training skill test scores among the 3 groups. Subsequent pairwise comparisons demonstrated that the tele-education group scored significantly higher than the self-directed learning group (*p* = 0.01), and so did the conventional education group compared to the self-directed learning group (*p* < 0.01). However, there was no statistically significant difference seen in scores between the tele-education and the conventional education groups (*p* = 0.56) (Fig. 4). There was no association between the skill test scores and the hours spent on pre-course materials (*p* = 0.33).

There were significant differences in improvement in participants’ subjective confidence levels in all machine operation, image acquisition, and image interpretation among the tele-education, the conventional education, and the self-directed learning groups. Subsequent pairwise comparisons demonstrated that compared to the self-directed learning group, both the tele-education and the conventional education groups had a significantly higher improvement in subjective confidence levels in machine operation (*p* < 0.01, *p* = 0.01, respectively) and image interpretation (*p* = 0.013, *p* < 0.01). A similar trend was observed in image acquisition (tele-education vs. self-directed learning, *p* < 0.01; conventional education vs. self-directed learning, *p* = 0.017). There were no significant differences in improvement in participants’ subjective confidence levels in any of the confidence categories between the tele-education and the conventional education groups.

The median [IQR] NASA-TLX score (maximum of 100) as a surrogate of subjective workload in the tele-education, the conventional education, and the self-directed learning groups was 54 [47–61], 57 [22–64], and 66 [63–72], respectively (*p* = 0.05). Although the higher median NSASA-TLX score in the self-directed learning suggested a higher perceived workload compared to the other 2 groups, no statistically significant difference was observed in the pairwise comparisons under a corrected alpha level; the tele-education vs. self-directed learning groups (*p* = 0.03), and the conventional education vs. self-directed learning groups (*p* = 0.04) (Fig. 4).

The median [IQR] participants’ satisfaction of the hands-on training in the tele-education, the conventional education, and the self-directed learning groups was 9 [9], 10 [8.5–10], and 6 [5–7] on a 10-point Likert scale, respectively (*p* < 0.01). The tele-education group was asked 2 additional questions in their post-training surveys (1 for strongly disagree, 5 for neutral, and 10 for strongly agree); Question No.1; Do you think that the quality of your ultrasound training would have been better if the instructor was present in the room? Question No.2; Do you agree or disagree with the following statement; I would recommend the remote ultrasound training to my colleagues? The median [IQR] rating was 4 [4-7.3] on question no.1 and 9 [8.5–10] on question no.2.

Narrative feedback from each group was as follows. From the tele-education group, “It was a great opportunity to learn the basics; however, it would have been useful to have a hands-on instruction when questions arise, rather than receiving verbal instructions remotely.” and “Not having an actual supervisor may decrease learners’ stress.” From the conventional education group, “It was nice to have guidance on hand positions and learn techniques to achieve the optimal ultrasound image.” and “It was easy to learn with an instructor present.” From the self-directed learning group, “It was difficult not to have feedback on how to improve techniques.” and “More time than allotted is needed to learn the ultrasound skill in the self-directed learning method.”

## Discussion

Our study demonstrated that our tele-education model, combining online didactic lectures and tele-mentored hands-on training was more effective than self-directed learning. There was no statistically significant difference in learners’ POCUS skill test scores between the tele-education and the conventional education groups. Improvement in the learners’ subjective confidence levels before and after the course was significantly higher in the tele-education group compared to the self-directed learning group, while no significant difference was observed between the tele-education and the conventional education groups. Tele-education did not result in a significantly higher perceived workload compared to conventional training or self-directed learning. The post-training survey revealed a similar satisfaction level of the tele-education experience to that of the conventional education, which was also supported by favorable narrative responses.

There has been a growing interest in remote ultrasonography training; however, most of published reports were proof-of-concept studies that mainly focused on the feasibility of the education platforms the study teams created. With a rapid technological advance and an increased need for robust tele-education systems delineated by the recent COVID-19 crisis, more sophisticated, all-in-one solutions are now commercially available. Indeed, recently published studies from the United States explored the effectiveness of a tele-ultrasound curriculum “Lumify with Reacts” (Phillips/Innovative Imaging Technologies) as an alternative to disrupted conventional in-person ultrasound training due to COVID-19 pandemic [27, 28]. In a non-inferiority study that included 56 novice learners, Drake et al. found no inferiority in the leaners’ ultrasound competency in the tele-ultrasound group compared to that in the traditional group when performing the predetermined tasks of the following 3 exams: focused assessment with sonography in trauma (FAST, 6 questions), lower extremities deep venous thrombosis screening (5 questions), and ultrasound-guided vascular access (3 questions) [27]. Soni et al. conducted a retrospective observational study in which pre and post-course knowledge test scores were compared between the tele-ultrasound (*n* = 52) and the traditional in-person (*n* = 88) cohorts in the continuing medical education course on POCUS [28]. The results indicated that the tele-ultrasound may be as effective as the conventional in-person course. Our study results are concordant with those recently published studies, which support the concept that tele-education model in ultrasound training is a reasonable alternative to conventional education.

There are a few unique elements in our study. First, prior to proceeding with a 3-arm randomized study, our study team spent a considerable amount of time launching a practical, yet HIPAA-compliant secure tele-communication system that could be applied not only to an education setting but also to a clinical practice. The most challenging part was to develop an audio-visual system configuration that would enable simultaneous live streaming of ultrasound images and verbal communications between a remote instructor and learners, since timely feedback was known to be one of the important elements of successful training [25, 31, 32], In the present study, live video inputs from the ultrasound machine and the webcam were integrated into the Vidyo interface in conjunction with the audio input from the speakerphone, allowing a successful tele-coaching platform on the remote instructor’s desktop computer with top-bottom dual-video feeds. Acquiring a speakerphone, a portable webcam, and an analog-to-digital video converter proved relatively inexpensive. However, the most substantial expense was the purchase of the software-specific codec. The entire system was configured to auto-activate as the ultrasound machine is turned on; therefore, it did not add surplus time or friction on starting the tele-education session. This practicality and accessibility from both learners’ and instructors’ perspectives significantly enhanced the quality of tele-coaching, which likely contributed to an improvement in learners’ ultrasound skill and their subjective confidence levels without posing significant perceived workload or cognitive stress in the tele-education group. Second, terminology used in verbal instructions during tele-education was standardized. All learners underwent a 30-minute orientation session via tele-conference immediately before their assigned training sessions. This orientation included an explanation of standardized verbal cues to guide the learner’s hand and transducer manipulation. For instance, an instructor used a clock position to point a transducer direction and “medial/lateral” to the standardized patient to guide the direction of the transducer sliding. This standardization likely avoided potential miscommunication between an instructor and learners in transducer handling and led to more effective and cognitively less stressful training. Finally, the present study design was unique in evaluating 3 different ultrasound education methods not only in performance measures but also in their quantified, perceived workload. In the field of medicine, the mental workload has been measured in the context of procedures, work environment, and system transition, most commonly with the use of NASA-TLX score [33–35]. Recently, there have been a few studies that utilized the NASA-TLX score as a surrogate of learner’s perceived workload in ultrasound education; [36, 37] however, our study is the first to specifically measure the workload of the learners who underwent tele-education focused on acquisition of POCUS skills.

Our study has several limitations. First, the study was not designed to test non-inferiority of tele-education to the conventional training. Second, only ultrasound novices were recruited for one-time ultrasound training to study their short-term skill improvement. The role of tele-education in intermediate or advanced learners or its long-term efficacy is unknown. None of the study participants had conducted POCUS in actual patient care or attended a regional or institutional POCUS workshop. However, participants’ pre-course familiarity with the ultrasound machine and their POCUS knowledge and skill sets might not have been entirely uniform. This variability may have confounded our study results. Third, time spent on pre-course materials was not standardized or monitored. Nevertheless, there was no association between the self-reported time spent on pre-course materials and scores in POCUS skill test. Fourth, the cognitive burdens of a remote instructor were not measured. A study suggested that a tele-ultrasound model posed a high cognitive burden on remote instructors [38], which is an important theme to be addressed in future studies.

In conclusion, our study demonstrated that a tele-education method, combining online didactic lectures and remote hands-on training was more effective than self-directed learning. There was no statistically significant difference in the effectiveness represented by POCUS skill test scores between the tele-education and the conventional education groups. Tele-education did not impose a significantly higher workload on learners compared to the conventional education or self-directed learning. Lastly, tele-education was well-accepted by leaners and has a substantial potential as an alternative POCUS education strategy.

## Electronic supplementary material

Below is the link to the electronic supplementary material.

Supplementary Material 1

Supplementary Material 2

## Data Availability

The datasets created and/or analyzed during the current study are available from the corresponding author on a reasonable request.

## Abbreviations

POCUS: Point-of-care ultrasonography
COVID-19: Coronavirus disease 2019
HIPAA: The Health Insurance Portability and Accountability Act of 1996
NASA-TLX: NASA Task Load Index
