# Supplementary material for: Tele-education in point-of-care ultrasound training
Source: Ultrasound J. 2024 Oct 28;16:47. doi: 10.1186/s13089-024-00394-1 (PMC11519237; doi:10.1186/s13089-024-00394-1)
Supplement: Supplementary file 2 — Supplementary Material 2 [file 13089_2024_394_MOESM2_ESM.pdf]

Supplementary Material 2: Skill Portion of the Critical Care Ultrasonography (CCUS) Proficiency Test

Scenarios:

The skill portion of the CCUS proficiency test consisted of 6 sections. An examinee was asked to complete the tasks outlined on the written instruction sheets within a set timeframe. A standardized patient, a healthy male volunteer, was utilized for the examination.

Instructions:

A. Ensure the target organ is **centered on the screen** with correct orientation.

B. **Save** video images with appropriate depth and gain settings.

C. Video recording is set to **a 6-second prospective mode**. The machine will record a 6-second video clip **after** pressing a button.

D. Position the patient appropriately, except for abdominal CCUS.

E. Do not turn off the machine until instructed to do so.

Section 1: Starting the Examination

You are to **initiate a CCUS examination.**

You have **3 minutes** to complete the task.

1. Power on the machine.
2. Manually enter or scan the patient's clinic number to start the examination. (No need to enter the patient's first and last name.)
3. Notify the examiner upon completion of the task.

Section 2: Vascular CCUS

You are to perform **a vascular CCUS examination** on a patient with a swollen right leg.

You have **4 minutes** to complete the task.

1. Visualize the **right common femoral vein and artery** (2 structures side by side). Ensure appropriate depth and gain settings.
2. Save a video clip while performing **a compression maneuver**.
3. Visualize the **right popliteal vein and artery**. Ensure appropriate depth and gain settings.
4. Save a video clip while performing **a compression maneuver.**
5. Notify the examiner upon completion of the task.

Section 3: Abdominal CCUS

You are to perform **an abdominal CCUS examination** on a patient with hypotension, abdominal pain, and anuria. The patient is supine and **immobile.**

You have **2 minutes** for probe switching and **4 minutes** to complete the task.

1. Visualize **the abdominal aorta** and save a video clip **in a longitudinal view**.
2. Visualize **the left kidney** and save a video clip **in a longitudinal view**.
3. Visualize **the gallbladder body (fundus)** and save a video clip **in a longitudinal view.**
4. Notify the examiner upon completion of the task.

Section 4: Thoracic CCUS

You are to perform **a thoracic CCUS examination** on a patient experiencing respiratory distress.

You have **3 minutes** to complete the task.

1. Perform a CCUS examination of **the right anterior chest** (**second or third intercostal space** in the midclavicular line) and save a video clip. Ensure appropriate depth and gain settings.
2. Visualize **the entire right hemidiaphragm** and save a video clip. Ensure appropriate depth and gain settings.
3. Notify the examiner upon completion of the task.

Section 5: Cardiac CCUS

You are to perform **a cardiac CCUS examination** on a hypotensive patient.

You have **2 minutes** for changing the examination mode and **6 minutes** to complete the task.

1. Obtain **a subcostal** (subxiphoid) **4 chamber view** and save a video clip.
2. Obtain **a subcostal** (subxiphoid) **inferior vena cava view** and save a video clip.
3. Obtain **a parasternal long-axis view** and save a video clip.
4. Obtain **a parasternal short-axis view at the papillary muscle level** and save a video clip.
5. Obtain **a parasternal short-axis view at the aortic valve level** and save a video clip.
6. Obtain **an apical 4-chamber view** (avoid foreshortening) and save a video clip.
7. Notify the examiner upon completion of the task.

Section 6: Ending the Examination

You are to **conclude the examination**.

You have **3 minutes** to complete the task.

1. Properly end the examination and power off the machine.
2. Wipe the ultrasound gel off the patient.

3. Clean the machine and probe.
4. Notify the examiner upon completion of the task.

Checklist

The CCUS skill checklist comprised 23 items, with a maximum raw score of 50. Knobology accounted for 14% of the total score, while cardiac contributed 38%, abdomen 18%, vascular 18%, and thoracic CCUS 12%. The entire skill test was videotaped, and the archived images were reviewed for subsequent scoring by an investigator (R.S.). If a participant recorded multiple images for one task, the reviewer scored the most preferable image.

For knobology and patient positioning, 1 point was given if the task was completed. For organ visualization, no point was given for no images or unidentifiable structures, 1 point for an inadequate image of the target organ, insufficient for answering a clinical question, 2 points for a suboptimal image of the target structure, yet acceptable for answering a clinical question, and 3 points for an optimal image in every aspect, including depth and gain, sufficient for answering a clinical question. A full 2 points were given for a complete compression maneuver; however, only 1 point was given for an inadequate compression.

Section	Clinical Questions	Checklist Items	Scores
Knobology	None	Power on the machine?	1
		Start a new examination and enter clinic number?	1
		Switch a transducer from vascular to abdomen?	1
		Switch an examination mode from abdomen (thoracic examination) to cardiac?	1
		End the examination before powering off the machine?	1
		Wipe ultrasound gel off the standardized patient?	1
		Clean the machine and transducers?	1
Abdomen	Aortic aneurysm, hydronephrosis, or cholecystitis?	Visualize the aorta?	3
		Visualize the left kidney?	3
		Visualize the gallbladder?	3
Thoracic	Pneumothorax or diaphragmatic dysfunction?	Visualize the anterior thorax?	3
		Visualize the right diaphragm?	3
Cardiac	Cardiomyopathy, valvulopathy, or abnormal central venous pressure?	Optimize patient position?	1
		Obtain a subcostal 4-chamber view?	3
		Obtain an inferior vena cava view?	3
		Obtain a parasternal long axis view?	3
		Obtain a parasternal short axis view at the mid papillary level?	3

		Obtain a parasternal short axis view at the aortic valve level?	3
		Obtain an apical 4-chamber view?	3
Vascular	Atherosclerosis or deep vein thrombosis?	Optimize patient position?	1
		Visualize the right common femoral vein and artery?	3
		Visualize the right popliteal vein and artery	3
		Perform compression maneuver?	2
Total			50
